# RECCIPE: A new framework assessing localized cell-cell interaction on gene expression in multicellular ST data

**DOI:** 10.3389/fgene.2024.1322886

**Published:** 2024-01-24

**Authors:** Weiping Ma, Xiaoyu Song, Guo-Cheng Yuan, Pei Wang

**Affiliations:** ^1^ Department of Genetics and Genomic Sciences, Icahn School of Medicine at Mount Sinai, New York, NY, United States; ^2^ Tisch Cancer Institute, Icahn School of Medicine at Mount Sinai, New York, NY, United States; ^3^ Institute for Health Care Delivery Science, Department of Population Health Science and Policy, Icahn School of Medicine at Mount Sinai, New York, NY, United States

**Keywords:** spatial transcriptome, spatial neighborhood, cell type proportion, cell-cell interaction, regression model, FDR control, Alzheimer disease

## Abstract

Cell-cell interaction (CCI) plays a pivotal role in cellular communication within the tissue microenvironment. The recent development of spatial transcriptomics (ST) technology and associated data analysis methods has empowered researchers to systematically investigate CCI. However, existing methods are tailored to single-cell resolution datasets, whereas the majority of ST platforms lack such resolution. Additionally, the detection of CCI through association screening based on ST data, which has complicated dependence structure, necessitates proper control of false discovery rates due to the multiple hypothesis testing issue in high dimensional spaces. To address these challenges, we introduce RECCIPE, a novel method designed for identifying cell signaling interactions across multiple cell types in spatial transcriptomic data. RECCIPE integrates gene expression data, spatial information and cell type composition in a multivariate regression framework, enabling genome-wide screening for changes in gene expression levels attributed to CCIs. We show that RECCIPE not only achieves high accuracy in simulated datasets but also provides new biological insights from real data obtained from a mouse model of Alzheimer’s disease (AD). Overall, our framework provides a useful tool for studying impact of cell-cell interactions on gene expression in multicellular systems.

## 1 Introduction

The biological function of an organ or tissue in a multicellular organism is the result of the concerted action of many cells, each carrying out their own specialized tasks. Understanding of the fundamental principles of how cells interact with each other would have a profound impact on knowing the molecular basis for disease initiation and progression. Powered by the recent development of spatial transcriptomics (ST) technologies, researchers can now simultaneously dissect the complex cell state-specific transcriptomic landscape while preserving the spatial context. As such, these technologies offer an unprecedented opportunity to comprehensively study the molecular mechanisms driving or being driven by interactions between cells and their tissue environment, a crucial aspect for unraveling developmental processes of various human diseases.

A key task in ST analysis is to identify gene expression changes relating to cell-cell interactions (CCI). While several methods have been previously developed (Browaeys, Saelens, and Saeys, 2020; Cang and Nie, 2020; Yuan and Bar-Joseph, 2020; Li et al., 2023), existing methods typically demand single-cell resolution. In contrast, common transcriptome-wide ST data generation platforms, such as Spatial Transcriptomics (Stahl et al., 2016), Visium by 10X Genomics (Maynard et al., 2021), Slide-seq (Rodriques et al., 2019), and DBiT-seq (Liu et al., 2020), do not have single-cell resolution. Consequently, there arises a necessity for innovative strategies capable of incorporating cell type composition information when assessing CCI within datasets produced by these platforms.

In addition, previous studies have primarily focused on identifying interactions between ligand-receptor pairs (Cang and Nie, 2020; Pham et al., 2020; Yuan and Bar-Joseph, 2020; Li et al., 2023). While the initial interaction between ligands and receptors serves as the primary mechanism through which one cell can influence another, the activated or inhibited receptor typically initiates a cascade of biological signaling events within the respective cell subsequently. The comprehensive gene profiles obtained from spatial transcriptomics (ST) data provide a valuable tool for characterizing not only the receptor itself but also the molecular changes downstream that are elicited by cell-cell interactions (CCI). For example, a recent study using single cell ST data (Browaeys, Saelens, and Saeys, 2020) successfully demonstrated the impact of ligand-receptor interaction patterns on downstream gene expressions, and showed that re-wiring of cellular signaling due to CCI can be cell type specific. As such, new methods are needed to support transcriptome-wide CCI analysis for datasets without single-cell resolution. A key challenge for transcriptome-wide analysis is the difficulty in controlling the false discovery rate (FDR) under the high dimensional space. While several established methods (Miller, 1966; Y; Benjamini and Hochberg, 1995; Benjamini and Yekutieli, 2005; Stephens, 2017) address this concern by modifying the significance of tests for multiple comparisons in various fields, these assumptions might not be applicable within the context of CCI analysis based on ST data. This is primarily due to the complicated correlation structure in the ST data, which results in severe collinearity among variables.

To overcome the aforementioned challenges, we propose a new CCI analysis pipeline: **RECCIPE**, short for the **RE**gression framework evaluating **CCI** on ST data within the context of s**P**ot n**E**ighborhoods). RECCIPE utilizes a multivariate regression framework to model the dependence of gene expression changes in each ST spot (grid) on the concentration of different cell types in their immediate neighbor regions, and at the same time accounting for the cell type composition’s heterogeneity within the ST spot. For estimating cell type composition, RECCIPE employs customized deconvolution tools tailored specifically to ST datasets. To screen for significant CCI-driven genes across the entire genome, RECCIPE employs a local false discovery rate (FDR) control method (Efron, 2007), which is capable of handling data with strong dependence structure.

RECCIPE can be applied to any ST dataset regardless of its spatial resolution. To demonstrate its efficacy, we conducted a comprehensive evaluation of RECCIPE’s performance using synthetic ST datasets. In addition, we applied RECCIPE to a ST dataset derived from mouse brains (W.T. Chen et al., 2020), and successfully identified CCIs that are unique to Alzheimer’s disease (AD) samples, underscoring its potential as a valuable tool in deciphering complex CCI from ST data.

## 2 Materials and methods

### 2.1 Overview of RECCIPE

**FIGURE 1:**
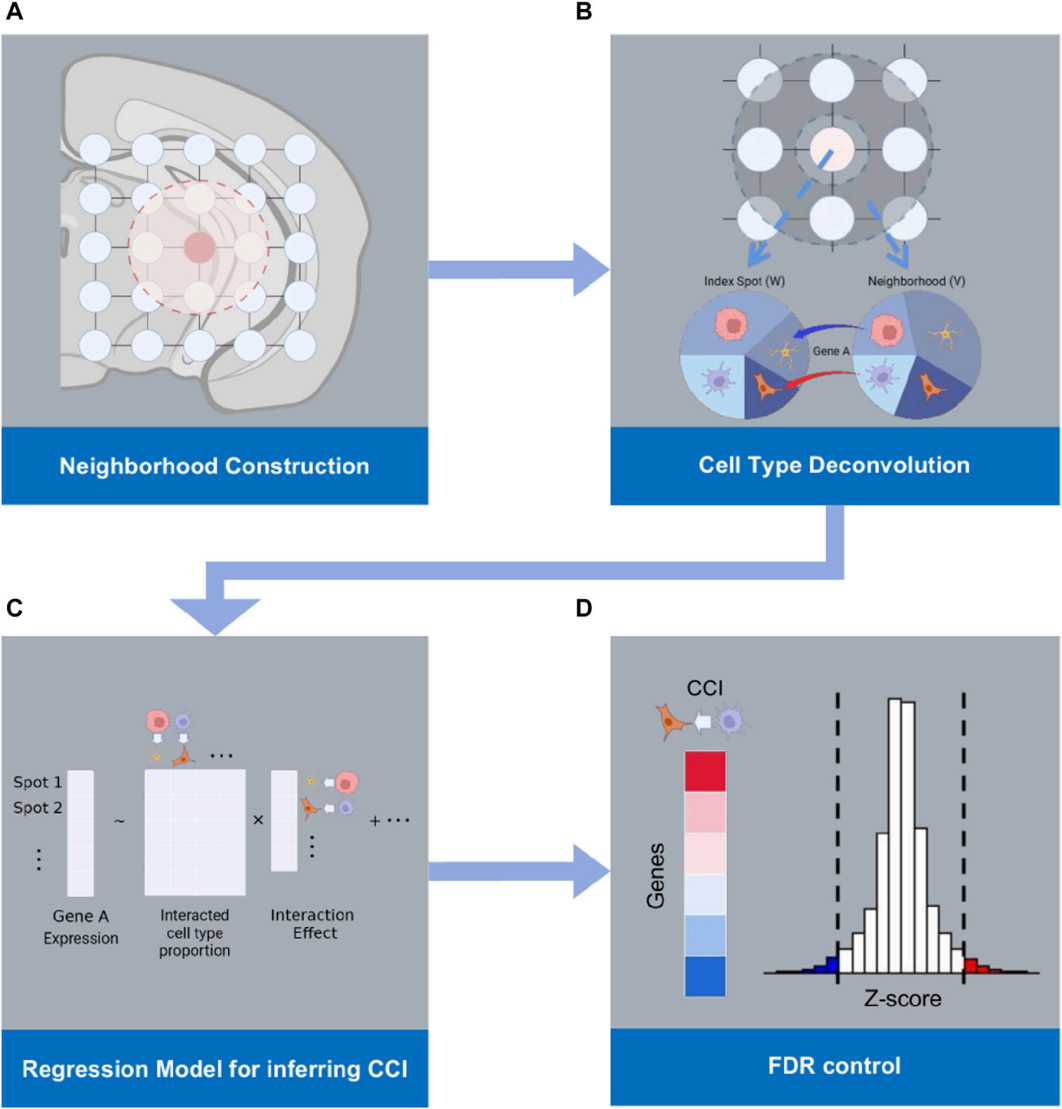
Workflow of RECCIPE: **(A)** RECCIPE starts from defining the physical neighborhood of each spot from ST data. **(B)** Cell type compositions of each spot and the corresponding neighboring region are derived from the ST data using the deconvolution method. **(C)** Regression models are used for screening for associations between the gene expression changes in a given spot and the percentages of different cell types in the spot and its neighborhood. **(D)** Transcriptome-wide screening for significant associations between gene expressions in the index spot and density of relevant cell type pairs are obtained with proper FDR control.

When CCI occurs between two distinct cell types, T_1_ and T_2_, it is expected that the gene expression within a cell of type T_1_ may exhibit up- or downregulation in response to an increased presence of cells of type T_2_ in its vicinity. RECCIPE is designed to identify these association patterns between gene expressions in one cell type and the concentrations of other cell types within their respective neighborhoods. The workflow of the RECCIPE pipeline is illustrated in Figure 1. Briefly, RECCIPE takes the ST dataset derived from one biological sample as input (Figure 1A). For each ST spot (grid) in the data set, RECCIPE firstly defines its immediate neighboring spots based on physical distances between spots on the tissue slice (Figure 1B). It then applies the spatial deconvolution method (spatialDWLS) (Dong and Yuan, 2021) to estimate the cell type proportions in the tissue spot. Consequently, cell type composition of the neighbor-region is calculated as the average of cell type compositions from all the spots in the neighbor-region. RECCIPE then screens for associations between gene expressions in one spot and the concentrations of different cell types in its neighbor-region using a multivariate regression framework (Figure 1C). In the end, RECCIPE determines the statistically significant gene—cell type associations by applying the local fdr control method (Efron, 2007) on estimates from all genes (Figure 1D). Below we elaborate these steps one by one.

### 2.2 Neighborhood identification and cell type decomposition

RECCIPE utilizes a Spatial Transcriptomic toolbox Giotto (Del Rossi et al., 2022) for the ST data preprocessing, including data normalization, neighborhood identification and cell type decomposition. Briefly, RECCIPE first normalizes expression matrix of the ST experiment using the standard approach in Giotto to remove technical variation across spots or genes. RECCIPE then defines the immediate neighbors of each spot through constructing a spatial network among all measured spots using Delaunay triangulation network (Del Rossi et al., 2022). The Delaunay network, which has been adopted in various fields of biology (Goltsev et al., 2018), provides a flexible selection of neighbors when the spots are not on the regular grids of the space. Neighborhood of one spot is then naturally defined as the spots directly connected to it (Figure 1A). Note that, when the spots are regularly distributed on the 2-dimensional slices (such as for 10X), this procedure is equivalent to defining neighbors using a distance cutoff. Moreover, RECCIPE applies the Spatial deconvolution method, spatialDWLS (Dong and Yuan, 2021), to estimate the cell type proportions in each tissue spot according to a cell type signature reference matrix. This method is an extension of dampened weighted least squares combining a recently developed cell-type enrichment analysis method (Del Rossi et al., 2022) to enhance specificity.

In the end, after the spatial neighborhood is identified and cell type compositions of each spot are estimated, RECCIPE further determines the cell type proportions for each neighborhood. Denote $w_{t}^{s}$ as the estimated proportion of cell type *t* in spot *s*, the proportion of cell type *t* for the neighborhood of spot *s* is then defined as the average of the proportions of this cell type across all spots in the neighborhood*:* ${\;v}_{s,t}=\frac{1}{\left|B_{s}\right|}\sum^{u\in B_{s}\;}w_{t}^{u}$ , where $B_{s}$ is the set of neighbors of spots.

### 2.3 Multiple regression model on CCI

In ST experiments conducted at the multi-cellular resolution, gene expression measurements of a single spot (grid) can be conceptualized as a weighted average of the gene expressions of individual cell types within that spot. These weights can be proportionally determined by the respective percentages of each cell type present in the spot. Denote the gene expression measurement of gene *g* in spot *s* as $Y_{s}^{g}$ , we have:

$$Y_{s}^{g}=\sum_{t\in T}w_{t}^{s}\times Y_{s,t}^{g},$$

where $Y_{s,t}^{g}$ denotes the expression value of gene *g* in cell type *t* within the spot *s*; $w_{t}^{s}$ is the proportion of cell type *t* within spot *s*; and T denotes the set of all possible cell types in the experiment.

Additionally, in the presence of CCI, the gene expression in a particular cell type t within the spot can be influenced by the presence or abundance of another cell type in the neighboring region of the spot (Figure 1). We then model $Y_{s,t}^{g}$ using the below equation:

$$Y_{s,t}^{g}=\beta_{t}^{g}+\sum_{tj\in T}^{tj!=t}\gamma_{t,tj}^{g}\times v_{s,tj}+{Ɛ}_{s,t}^{g},$$ (1)

where $\beta_{t}^{g}$ denotes the mean expression level of gene *g* in cell type *t*; $v_{s,tj}$ is the proportion of cell type *tj* in the neighborhood around spot *s*; and $\gamma_{t,tj}^{g}$ represents the strength of the CCI influence from cell type *tj* on gene *g* in cell type *t*. Combine the above two equations, we have:

$$Y_{s}^{g}=\sum_{t\in T}\beta_{t}^{g}\times w_{t}^{s}+\sum_{t\in T}\sum_{tj\in T\;}^{tj!=t}\gamma_{t,tj}^{g}\times v_{s,tj}\times w_{t}^{s}+e_{s}^{g},$$ (2)

where $e_{s}^{g}$ is an independent random error term.

Note, since $\sum v_{s,tj}$ = 1, to ensure the model identifiability, we chose to keep the intercept term but omit the $v_{s,t}$ term in Equation 1. This decision aims to prevent the simultaneous inclusion of both the $w_{t}^{s}$ and $v_{s,t}\times w_{t}^{s}$ terms in model (2). Because $w_{t}^{s}$ and $v_{s,t}\times w_{t}^{s}$ are derived from multicellular ST data, commonly exhibit high correlation, the inclusion of both terms could result in substantial variation in the coefficient estimates. For example, based on the real datasets analyzed in the paper, $w_{t}^{s}$ and $v_{s,t}\times w_{t}^{s}$ exhibiting an average Spearman correlation of 0.86 (sd = 0.038). This high collinearity greatly hampers the power to detect dependence of gene expressions on neighborhood cell type proportion ( $v_{s,t}$ ), which is an intrinsic limitation of multicellular ST data. To prevent singularity due to high collinearity, we opted to focus on detecting CCIs between different cell types, thereby excluding the term $v_{s,t}\times w_{t}^{s}$ from the RECCIPE regression model. This strategic exclusion aims to enhance the stability of the coefficient estimates.

In RECCIPE, we fit the above linear regression model for each gene using data from all the spots and obtained the estimates of the CCI effect { $\gamma_{t,tj}^{g}$ } for all possible pair of ( t,tj ).

### 2.4 Controlling false discovery rate with *Locfdr*

Based on the above model, detecting biologically meaningful CCI corresponds to identifying nonzero estimates of $\gamma_{t,tj}^{g}$ . Since ST data usually involves thousands or tens of thousands of genes, proper FDR control for the inference of { $\gamma_{t,tj}^{g}$ } is crucial. Particularly, the independence or weak-dependence assumptions used by many existing multiple comparison correction methods, such as BH (Y. Benjamini and Hochberg, 1995) and FWER (Miller, 1966), do not apply here. This is because, not only ST expression data { $Y_{s}^{g}$ } has complicated correlation structure, the cell type composition matrix of spots (W = { $w_{t}^{s}$ }) and neighborhoods (V = { $v_{tj}^{s}$ }) also present high correlations. Thus, the estimates of { $\gamma_{t,tj}^{g}$ } possess a complex/strong dependence structure, which poses challenges in proper FDR control.

To tackle this challenge, we adopted an empirical Bayesian based local fdr adjustment method: *Locfdr* (Efron, 2007) (R package: locfdr). Specifically, for a given pair of source cell type *tj* and target cell type *t*, we apply *Locfdr* to the test statistics of $\gamma_{t,tj}^{g}$ for all genes from all the regression models. Since theoretical null distribution is often distorted in the presence of high dependence structure, *Locfdr* re-calibrates the null distribution based on the empirical distribution of the test statistics (Efron, 2007). Briefly, *Locfdr* assumes that the majority in the middle sector of the empirical distribution of the test statistics should come from the null component. It then employs a parametric fit (i.e., Gaussian) to derive the calibrated null distribution based on the selected middle sector. Consequently, in the tail region, FDR can be estimated using the ratio between the density function of the calibrated null and that of the observed test statistics across all genes. As illustrated in our simulation experiments (next section 3.1), this strategy effectively controls the FDR at the targeted level.

### 2.5 Reference data sets used in the simulation experiments.

We employed a ST data set of mouse cortex obtained through seqFISH + technology (Eng et al., 2019) as a reference for our simulation experiments. In this dataset, gene expressions were measured for 523 cells of 12 cell types from 5 closely situated *fields of views* (FOV). The expression data contains 10 k genes across all cells. To prevent computational instability issues arising from the minority cell types with extremely low cell counts, we grouped the 5 cell types with the smallest cell numbers into a single category.

## 3 Results

### 3.1 Simulation experiment

#### 3.1.1 Synthetic data generation

Synthetic datasets were generated to assess the performance of RECCIPE. We started with simulating single-cell-level ST data and subsequently aggregated cells within the neighborhood to generate spot-level multicellular ST data. Single-cell location were specified by adding Gaussian perturbations in both the horizontal and vertical directions to equally spaced grids on squared slides. To assess the influence of tissue architecture and ST data resolution on RECCIPE’s performance, we examined various combinations of different domain structure settings and spot sizes.

**FIGURE 2:**
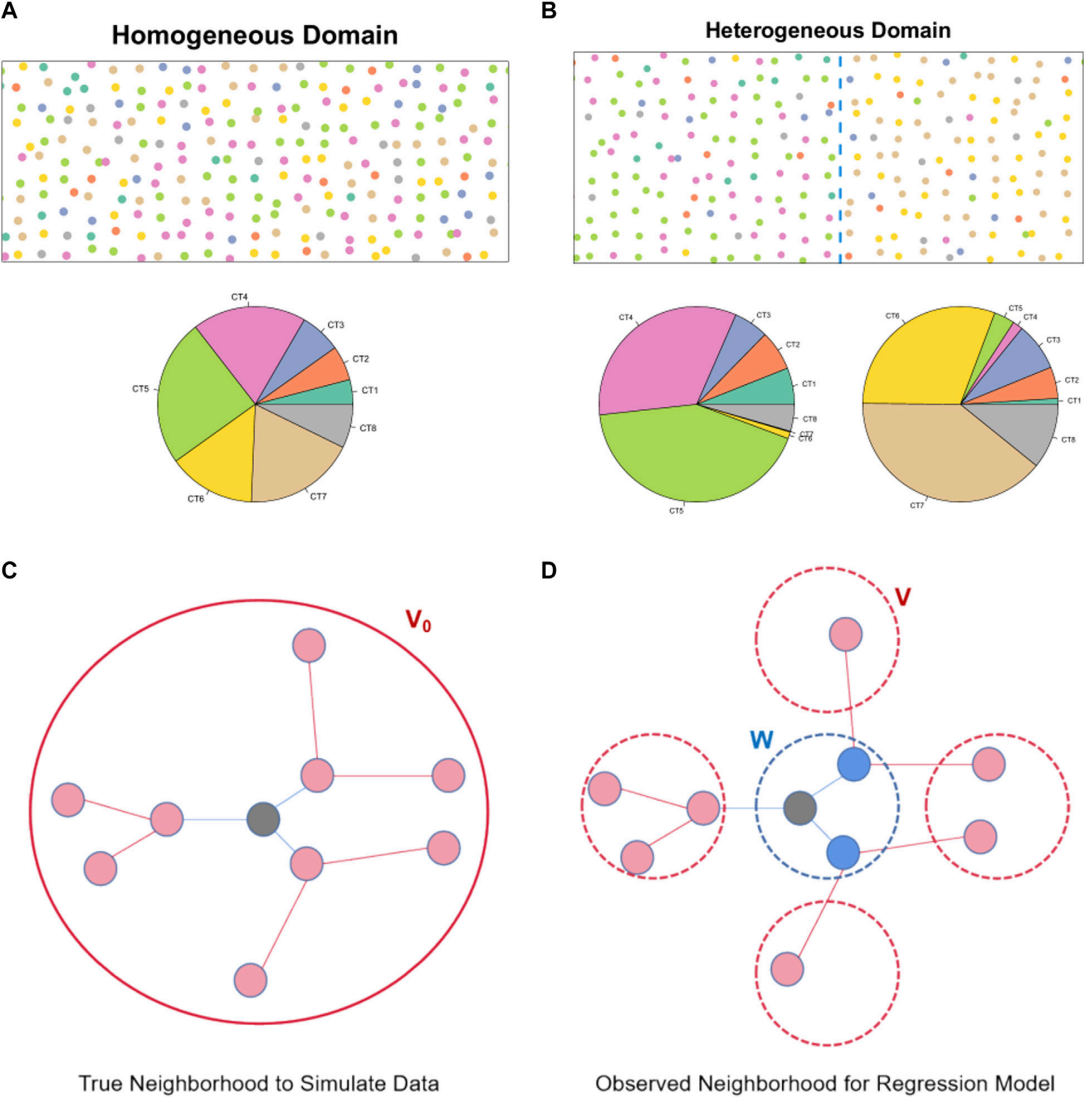
Cell type and spatial neighborhood generation in the simulation study: **(A)** An illustration of the spatial distribution of different cell types in the simulated cell-level ST data under the homogeneous simulation setting. The pie plot further summarizes the cell type proportions. **(B)** An illustration of the spatial distribution of different cell types in the simulated cell-level ST data under the heterogeneous simulation setting. The pie plots further summarize the cell type proportions in the two different domains. **(C)** An illustration of the index cell and its neighborhood region in the simulation. **(D)** An illustration of the index spot and its neighborhood region after cell-level data is aggregated to the spot-level.

We implemented two domain structures with different spatial arrangements of cell types: a homogeneous case and a heterogeneous case. In the homogeneous setup (Figure 2A), cell types were randomly assigned across the entire tissue slice, utilizing proportions derived from a publicly available seqFISH + dataset of mouse brain which has single-cell resolution (Eng et al., 2019) (see Section 2.5). In the heterogeneous case (Figure 2B), the tissue space was divided into two sections of equal size. Cell types were randomly generated within each section, adjusting the proportions based on estimates from the single-cell resolution ST data (Eng et al., 2019). The specific cell type proportions utilized in the simulation can be found in the (Supplementary Table S1).

When cells were grouped into pseudo spots, we considered different spot sizes to mimic different resolutions of ST technologies. Specifically, we designed a high-resolution setting approximating spot size of 55 μm in Visium experiments by 10X Genomics, and a lower-resolution setting for 100 μm in Spatial Transcriptomics (Stahl et al., 2016) (Supplementary Table S2). We ensured that the variability of cell numbers per spot in the simulated data closely mirrors that in the real datasets, as demonstrated in Supplementary Figure S1. Note, we leveraged the same seqFISH + dataset (Eng et al., 2019) to evaluate the variability in cell numbers across ST spots of either 55 or 100 μm (Stahl et al., 2016). In addition, when aggregating cells into pseudo spots, we organized the spots using regularly spaced grids both horizontally and vertically with prespecified distances.

To generate gene expression in each cell, we first derived mean expression profiles of 10,000 genes for each cell type using the aforementioned seqFISH + dataset (Eng et al., 2019). Then, we introduced CCIs effects for 500 randomly selected genes using specific combinations of source and target cell type pairs, as shown in Supplementary Table S3. The interaction effect values were determined by the product of randomly signed Gaussian effect sizes ( $\gamma_{t,tj}^{g}$ ) and the proportion of the source cell type ( $v_{s,tj}^{0}$ ) within the neighborhood. We considered second-degree-neighborhood in the Delaunay network in the high-resolution setting (Figure 2C, corresponding to spot size of 55 μm), and fourth-degree-neighborhood in the low-resolution setting (corresponding to spot size of 100 μm). Probabilities of positive and negative effect sizes were set to be equal (1⁄2). To emulate co-expressed patterns among genes, correlated Gaussian residuals were simulated for each cell. The correlation pattern was determined based on the estimated correlation matrix from the real data (Eng et al., 2019). As a result, single-cell gene expression profiles were synthesized by summing three components: cell type mean expression profiles, CCI effects, and the correlated residuals, as indicated in Eq. 1. In the end, based on the cell membership to pseudo spots, we aggregated the cell-level expression values to generate the spot-level expression profiles.

Furthermore, to understand the performance of the proposed regression framework on CCI signal strength, we considered different signal-to-noise ratio (SNR) levels in the above simulation. Here, SNR refers to the ratio of variance of CCI terms vs. the variance of the total expressions ( $Y_{s}^{g}$ ). We adjusted the size of CCI coefficients ( $\gamma_{t,tj}^{g}$ ) to control the final SNR at different levels (0.25, 0.5, 1, and 1.5).

In addition, to assess the robustness of RECCIPE to the violation of the working model specified in Eqs 1, 2, for each simulation setting on Slide 1, we considered two variations for specifying the CCI effects. Specifically, instead of using cell type proportions in the spot neighborhood ( $v_{s,tj}^{0}$ ) in Eq. 1, we used the count of cells of type tj in the spot neighborhood or the log-transformed cell count.

RECCIPE was then applied to the various gene expression matrices of pseudo spots to identify cell-cell interactions. Note, while $w_{s,tj}^{0}$ and $v_{s,tj}^{0}$ (Figure 2C) was used in the simulation to generate CCI effects, the estimates of $w_{s,tj}$ and $v_{s,tj}$ based on the spot-level data could be biased due to the reduced spatial resolution of spots/grids compared to single cells (Figure 2D). This was to mimic the challenging situation in the real ST data sets.

#### 3.1.2 Multiple testing correction methods for comparison

In the RECCIPE model, the total number of parameters of interest { $\gamma_{t,tj}^{g}$ } is |G|x (|T|-1)^2^, where |G| is the total number of genes and |T| is the total number of cell types considered in the analysis. As mentioned earlier, FDR control is a challenging task here due to both the large number of tests and complicated correlation structures among the ST data. Thus, in the simulation experiment, we benchmarked the performance of using *Locfdr* vs. other popular multiple hypothesis testing adjustment approaches in the RECCIPE framework. Specifically, we considered Bonferroni correction, Benjamini–Hochberg (BH) adjustment, Benjamini-Yekutieli (BY) procedure and a more recently developed empirical Bayesian correction method called “ash” (Stephens, 2017). Bonferroni correction aims to control the family-wise error rate (FWER), which involves multiplying the *p*-value of each individual test by the total number of tests (Miller, 1966). The Benjamini–Hochberg (BH) correction, a popular FDR control strategy, assumes all the testing to be independent (Y. Benjamini and Hochberg, 1995). The Benjamini-Yekutieli (BY) procedure is a variation of the BH method but can accommodate some (arbitrary) dependence among different tests (Benjamini and Yekutieli, 2005). In the end, the “ash” method employs an empirical Bayes approach and utilizes effect sizes and their standard errors from the test instead of marginal *p*-values for adjustment (Stephens, 2017).

In each simulation setting, we applied all aforementioned FDR control methods separately to identify CCI markers and summarized the performance accordingly.

#### 3.1.3 Performance evaluation in simulated data

To evaluate the performance, we assessed the power and true FDR for the CCI detection at a targeted FDR level of 0.1. Power was defined by the proportion of true CCI pairs (gene A in cell type *t1* vs. cell type *t2*) being identified by the model, while true FDR was the proportion of falsely detected signals by the model.

**FIGURE 3:**
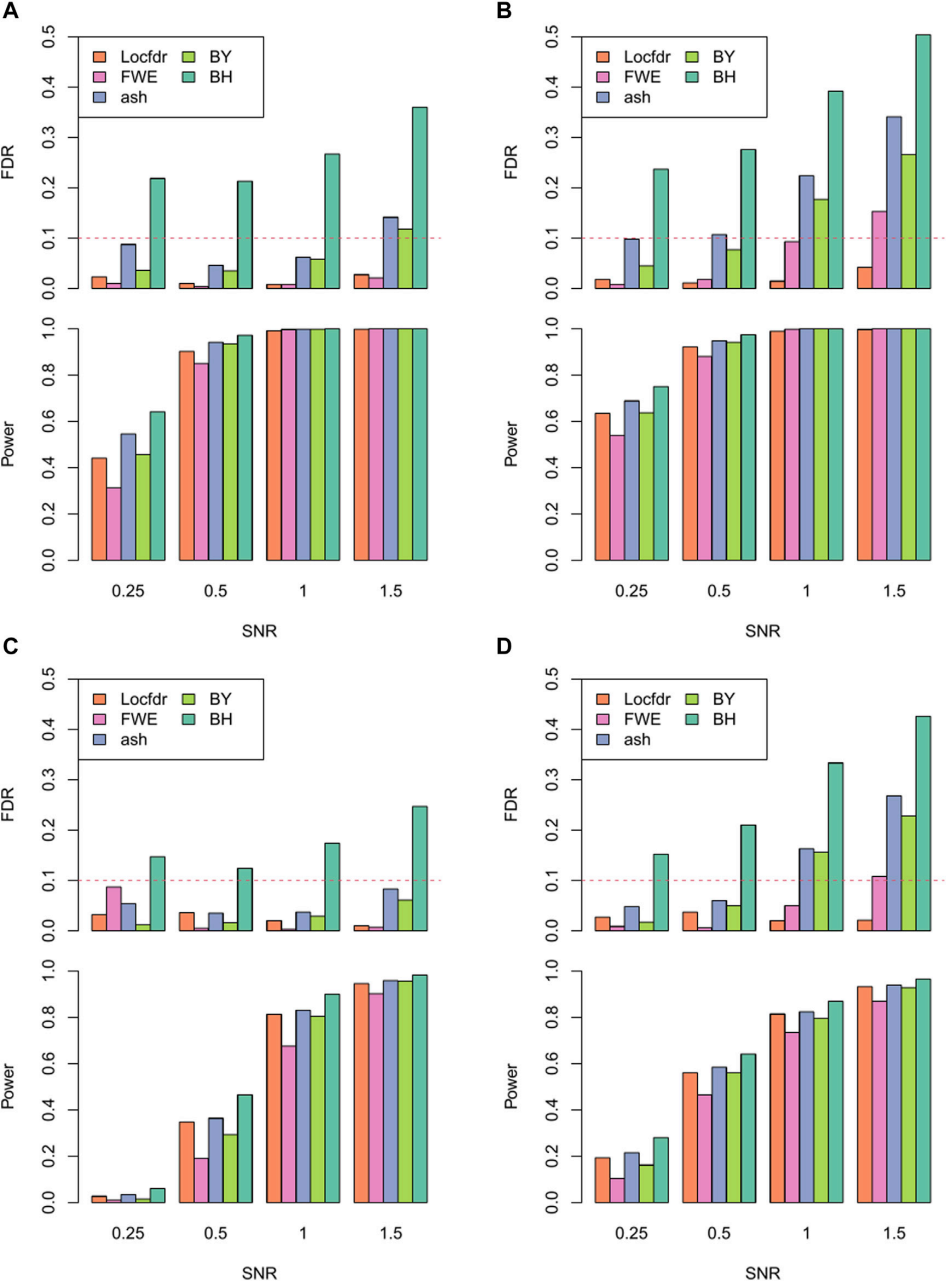
Power and FDR of different multiple comparison correction methods across different SNR levels under the combination of resolution and domain heterogeneity: **(A)**. High resolution in homogeneous domain. **(B)**. High resolution in heterogeneous domain. **(C)**. Low resolution in homogeneous domain. **(D)**. Low resolution in heterogeneous domain.

The performance of RECCIPE under various simulation settings is presented in Figure 3. Firstly, *Locfdr* is the only method that successfully controls the false detections below the targeted FDR level across all the simulation settings (Figure 3). All other methods showed flawed FDR controls, with BH displaying the most inflated FDR across all simulation settings. Interestingly, except for *Locfdr*, there exists an increasing trend in the (inflated) type-I error rates of the FDR correction methods as the SNR increases. This suggests the presence of groups of (false) features that are highly correlated with the true signals, which may be attributed to the extensive inter-gene and spatial correlations inherent in the ST data.

Moreover, when comparing results between the heterogeneous and homogeneous settings, most of the correction methods exhibit worse fdr control in the data sets from the homogeneous simulation. This observation implies that, when analyzing data from real tissue sections characterized by heterogeneous distribution of local cell type proportions, the task of maintaining FDR control in CCI screening could be considerably more challenging. This underscores the necessity of employing appropriate inference strategies, such as the one proposed here.

As to the power assessment, as expected, the power of RECCIPE increases with the SNRs (Figure 3). Especially, for the high-resolution setting, when the SNR is 0.5 or higher, the power of RECCIPE-*Locfdr* is over 90% under both the homogeneous and heterogeneous settings. In the contrast, the power of RECCIP on the low-resolution data has a 28% drop on average. This change is partially due to the decrease of sample size of spots in the low-resolution data.

**FIGURE 4:**
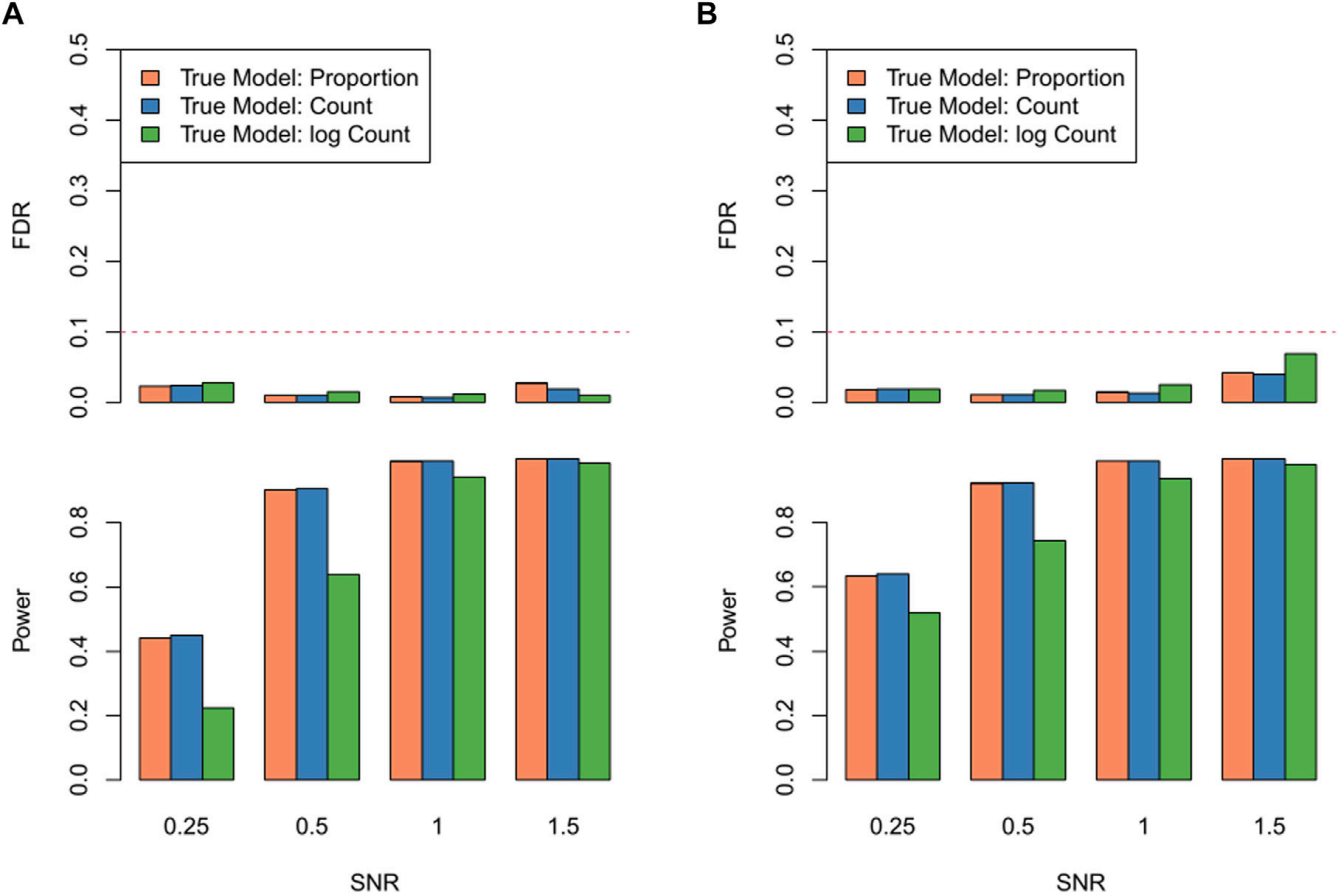
FDR and power of RECCIPE on high-resolution datasets with different signal generation settings across different SNR levels under the homogeneous setting **(A)** and the heterogeneous setting **(B)**.

In the exercise investigating robustness of RECCIPE against violations of model assumptions, the proposed model (RECCIPE-*Locfdr*) successfully controlled FDR across various settings (Figure 4). When the CCI effects were simulated using cell counts instead of the cell type proportion in the neighborhood, the powers of RECCIPE (blue bars) are comparable to that based on the correct model (orange bars) (Figures 4A,B). However, if the CCI effects were simulated based on log cell count (green bars), the power is about ∼20% lower than that based on the correct model (Figures 4A,B). Note, obtaining precise estimates of cell numbers from multicellular ST data poses a significant challenge. In addressing this challenge within the RECCIPE framework, we chose to utilize cell proportion estimates in the regression models. The results presented above suggest that this strategic choice provides sensible and robust results, even in the face of model mis-specification.

### 3.2 Real data analysis

We applied RECCIPE to an ST dataset of mouse brain on Alzheimer Disease (AD) (W.T. Chen et al., 2020).

#### 3.2.1 ST data, cell type deconvolution and neighborhood construction

**FIGURE 5:**
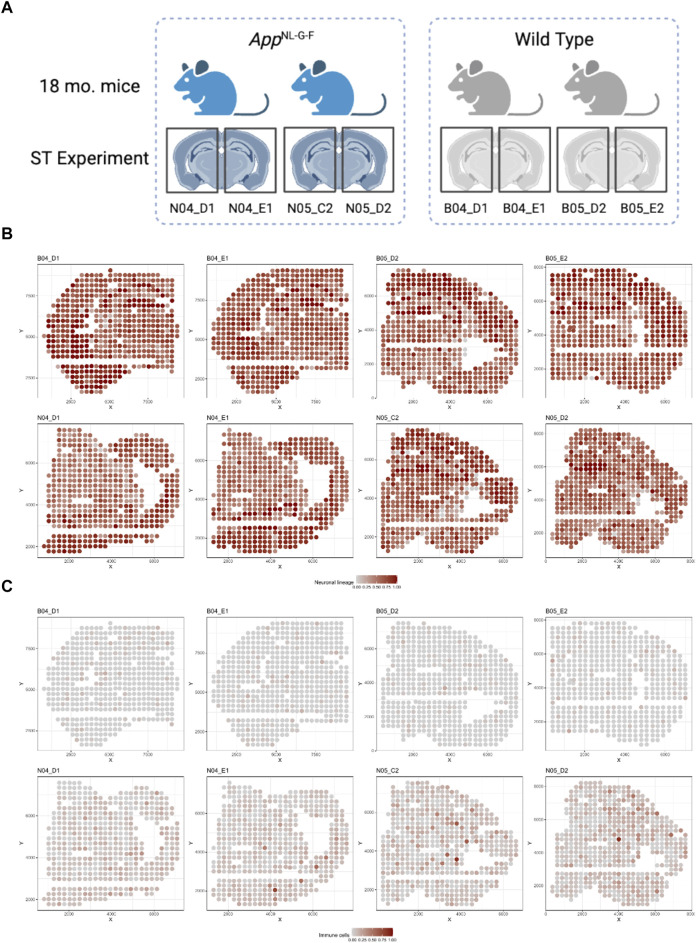
Illustration of the mice ST data sets: **(A)** Illustration for the design of the ST experiment on mouse brain with two groups (AD and WT) (W.T. Chen et al., 2020). **(B)** Spatial distribution for Neuronal lineage proportion of each spot among 8 samples. **(C)** Spatial distribution for Immune cell proportion of each spot among 8 samples.

ST datasets were collected for 8 brain samples from 4 mice at 18-month-old age: 2 wild type (WT) and 2 AD mice (Figure 5A) (W.T. Chen et al., 2020) via Spatial Transcriptomics platform (Stahl et al., 2016). Each ST dataset contains 510 spots on average, and the number of identified genes range from 36,863 to 38,047.

We used Giotto (Del Rossi et al., 2022), an integrative analysis and visualization tool for ST data analysis, to preprocess the mice ST data sets. Specifically, we applied a standard approach from Giotto: filtering out low expressed genes (on less than 50 spots) and spots (with less than 1,000 genes); normalizing the expression values on each spot with a scale factor of 5000 UMI counts. Scaled values of the normalized UMI counts were used as the expression matrix input of RECCIPE to detect CCI for each sample. After filtering, the average number of genes of each sample decreased to 12,701.

We then applied the spatialDWLS deconvolution method to estimate the cell type proportions in each spot using the Giotto package (Del Rossi et al., 2022). Specifically, since there was no single cell transcriptomic data set accompanied with the ST experiment for the cell type registration, we used the default cell type reference matrix provided by the Giotto package. Signatures from 21 distinct cell types were included. Cell type proportion estimates from spatialDWLS were further aggregated to 5 major cell types (see Supplementary Table S4 for detailed cell type group definition). Deconvolution results were displayed in Figures 5B,C and Supplementary Figure S2. Most of the cell types are distributed similarly across different samples. Neuronal cells were the dominant cell type across most spots in all 8 samples, particularly in the isocortex region (Figure 5B). Astro-ependymal cells and oligodendrocytes located mainly in the brain stem region in all samples (Supplementary Figure S2). However, immune cells were enriched in AD samples compared with WT samples (Figure 5C), which is consistent with the mechanism of immune infiltration in AD disease (Jorfi, Maaser-Hecker, and Tanzi, 2023).

To define the neighborhood of each spot, we constructed the Delaunay network of spots based on their physical locations on the tissue slices. Then for each spot, we defined its neighborhood as the spots directly connecting to it in the Delaunay network. Cell type proportions of each neighborhood region were then derived by averaging the cell type percentages of individual spots in the region. The design matrix for the regression models in RECCIPE was then specified based on cell type proportions in individual spots (W) and their neighborhood regions (V).

#### 3.2.2 Identify cell-cell interaction in the mouse brain

**FIGURE 6:**
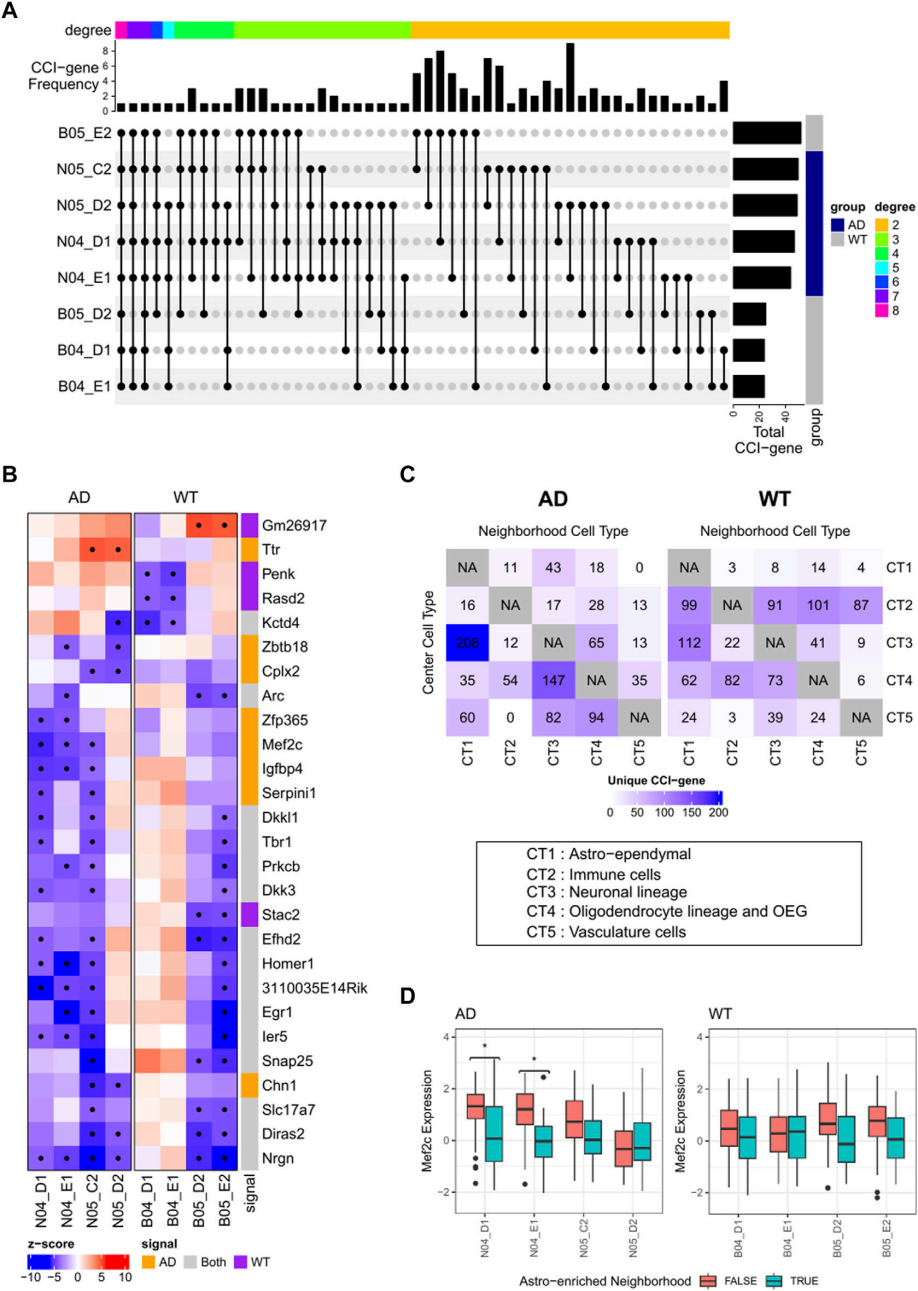
**(A)** Plot of identified CCI marker genes and the intersection among samples. Markers with at least 2 identifications across 8 samples are included. CCI Categories by combination of target and source are annotated on the top. **(B)** Heatmaps illustrating associations between gene expressions of selected genes in neuronal cells and cell type proportions of Astro-ependymal cells in the neighborhood. Significant associations are marked with dots in the heatmap. **(C)** Distribution of uniquely identified CCI-genes in AD or WT samples. Number of CCI-genes was labeled in the location with corresponding center spot cell type vs. neighborhood cell type. **(D)** Boxplots showing the distributions of Mef2c expressions in neuron-abundant spots (>75%) stratified by the percentage of Astro-ependymal cell types (dichotomized by 18%) in its neighborhood. Significant contrasts (FWER <0.1) are annotated with “*” in the plot.

Finally, we applied RECCIPE-*Locfdr* to each of the 8 ST datasets separately. Significant CCI pairs were declared at an FDR cutoff of 0.1. We referred to a gene as a CCI-gene (for a given sample) if the gene belongs to one of the significant CCI pairs identified for that sample. On average, we detected 240 CCI-genes per sample (Supplementary Table S5, Figure 6A). The CCI cell-type pair with the highest number of interactions are Astro-Neuron: 18.6% of the CCI-genes exhibited expression changes in neurons in response to alterations in the percentages of Astro-ependymal cells within their neighborhood. In addition, 29 out of the 240 CCI-genes were detected in more than one WT sample, while 58 CCI-genes were detected in more than one AD sample (Figure 6A). These occurrences were significantly higher than those from random selected genes with *p*-value <0.00001 (by permutation test of 100,000 replicates).

Detected CCI-genes was showing different distribution between AD and WT groups (Figure 6C), we then focused on the clear patterns differing between the two groups. We selected AD-specific or WT specific CCI-genes by screening for signals identified in at least 2 samples in one group, while totally absent in the other group. The group-specific markers were mainly related to Astro-Neuron CCI, including 8 AD-specific CCI-genes and 4 WT-specific CCI-genes Figures 6A,B. Additionally, 3 AD-specific CCI-genes associated with the Oligodendrocyte-Neuron CCI, and 4 AD-specific CCI-genes associated with the Neuron-Astro CCI (Figure 6A).

Among the AD-specific Astro-Neuron CCI markers, Mef2c exhibited high consistency across different samples. Specifically, in 3 out of the 4 AD samples, both genes exhibited decreased expressions in neuronal cells when the density of Astro-ependymal cells increased in the neighboring areas (Figure 6B). These associations were further illustrated in Figure 6D, which shows the distributions of Mef2c expressions in spots predominantly composed of neurons (with neuron cell percentage >75%), both with or without enriched Astro-ependymal cells (>18%) in the neighborhood. The enrichment cutoff at 18% is determined by the median value of Astro-ependymal cell proportions among the neighborhoods of all neuron-predominant spots for all samples. Notably, while significant mean shifts were observed in AD group (2 samples have FWER <0.1, FWER stands for Family Wise Error Rate), this pattern was absent in the WT samples (all FWER >0.1). The FWER was calculated by multiplying the *t*-test *p*-value with the total gene number. Interestingly Mef2c, as a gene known for its high expression in the nervous system, plays a significant role in neuron-development and inflammation regulation (Ren et al., 2022). Recent research has underscored its neuron-protective function in AD (Ren et al., 2022) and identified it as an key player for the epigenetic impact on the neuron-glial cross talk in AD (Zhou et al., 2023). Our analysis further suggests that Mef2c in neuron cells might be downregulated through the interaction between Astro-ependymal cells and neuron cells in brain tissue, which shed light on potential mechanisms contributing to the etiology of AD disease.

Other AD-specific Astro-Neuron CCI markers have also been reported to be relevant to AD. For example, Igfbp4 plays a crucial role as a neuronal survival factor (Son et al., 2019) and has been identified as a senescence marker of astrocytes, linked to AD progression (Carvalho et al., 2023). The chemerin/CMKLR1 axis participates in microglia migration and recruitment to senile plaques, potentially offering a new avenue for AD therapy (Y. Chen et al., 2022). Neuroserpin/Serpini1, a key tissue plasminogen activator (tPA) inhibitor in the brain, is upregulated in AD (Subhadra, Schaller, and Seeds, 2013), and its polymerization is implicated in human dementia (Davis et al., 1999). Cplx2-null mutant mice exhibit cognitive function loss in conjunction with a minor brain lesion, representing a relevant environmental risk (“second hit”) for schizophrenia (Begemann et al., 2010). Zbtb18 is essential for cerebellum growth, patterning, and neuron development (Baubet et al., 2012), and *de novo* variants in ZBTB18 are linked to intellectual disability (Cohen et al., 2017). TTR, known for neuroprotection in AD, is the primary Aβ binding protein in cerebrospinal fluid, naturally preventing Aβ aggregation and toxicity (Cotrina et al., 2021). Experimental evidence also suggests TTR’s role as a neuron-derived energy metabolism activator in astrocytes (Zawislak et al., 2017). These results confirmed that RECCIPE revealed biological and disease relevant CCI signals from the ST data.

## 4 Discussion

ST experiments conducted at spot resolution lack the granularity of individual cell information, making it challenging to explore interactions between cells or cell types. To facilitate the CCI analysis based on ST datasets without single-cell resolution, we propose a new method called RECCIPE, which employs a multivariate regression framework coupled with local FDR adjustment to conduct transcriptome-wide screening for CCI. RECCIPE utilizes a novel pipeline to extract cell type specific CCI effects by effectively integrating both the spatial information and cell type composition estimates from deconvolution analysis based on the spot-level ST data. Moreover, RECCIPE directly takes the (bulk) expression data of individual spots as inputs to the regression models and avoids performing a super resolution step to partition each spot. This not only streamlines the analysis process but also prevents the introduction of additional variation during inferences. We demonstrated the favorable performance of RECCIPE through extensive simulation experiments. When applied to AD-mice brain ST data, RECCIPE successfully identified biologically relevant CCIs among neuron and other cell types.

Transcriptome-wide screening for CCI involves an extensive number of tests, which posts difficulty on controlling FDR while maintaining robust power. This challenge is due to not only inherent correlation among gene expressions, but also the strong dependency across cell type proportions in neighboring spots. Through comprehensive investigations conducted on synthetic datasets, we elucidated the issue of FDR inflation associated with many commonly used FDR adjustment methods that assume independence or weak dependence among variables. Specifically, the assumption of FWER control on the approximation of combined *p*-values with Bonferroni inequalities (Miller, 1966), is only valid with a limited number of independent tests. Consequently, when screening CCI across an extremely large number of genes based on complicated ST data, FWER showed a lack of power on the synthetic data sets with low signal strength, while inflated type-I errors under settings of high SNRs. BH correction (Y. Benjamini and Hochberg, 1995) and its variation BY (Benjamini and Yekutieli, 2005) are both using a linear rejection line to relate the quantiles of the observed distribution of test statistics to that of the expected distribution under the null hypothesis. Both methods can only tolerate weak dependencies among tests, and thus result in many false signals in the proposed CCI analysis. The empirical Bayesian approach “ash” (Stephens, 2017) also suffers from inflated type-I error in our investigation, due to the violation of the underlying assumption of a unimodal distribution of the unobserved effect. In contrast, the simulation results provide compelling evidence of the effectiveness of the selected local FDR estimation approach, *Locfdr*, in successfully controlling the false discovery rate. Briefly, *Locfdr* is a data driven approach for estimating false discovery rates in large-scale hypothesis testing problems with either no/weak or strong correlations (Efron, 2007). This approach shows better performance in CCI screening compared to other methods across all simulation settings we considered.

To illustrate the application of RECCIPE, we applied it to ST datasets from a mouse study focusing on Alzheimer’s disease (AD) (W.T. Chen et al., 2020). Upon the successful identification of CCIs within each of the 8 samples (comprising 4 AD and 4 WT mice brain samples), we observed significant overlapping in the detected CCIs across different samples. In addition, a subset of CCIs were consistently detected in the AD samples while being notably absent in the WT samples. Importantly, many of the genes implicated in these CCI interactions were previously linked to AD in the scientific literature (Ren et al., 2022; Zhou et al., 2023; Son et al., 2019; Carvalho et al., 2023; Y; Chen et al., 2022; Subhadra, Schaller, and Seeds, 2013; Davis et al., 1999; Begemann et al., 2010; Baubet et al., 2012; Cohen et al., 2017; Cotrina et al., 2021; Zawislak et al., 2017). These findings collectively reinforce the credibility of the proposed CCI analysis through RECCIPE, and signify meaningful intercellular communication between various cell types within the context of AD.

Note, ST data of different biological samples often present non-negligible batch effects arising from technique variations in different ST experiment runs. To get around of this issue, in our analysis, we adopted a strategy of deriving cell-cell interactions (CCIs) for each biological sample (ST dataset) individually, followed by a joint interpretation of the inference results. This approach ensures that distinct technique variations in different ST experiments do not confound the CCI inference in the RECCIPE model. By doing so, the common and unique CCIs identified across different samples in WT mice or AD mice can more accurately reflect biological heterogeneity.

Distinguished from current CCI analysis on ligand-receptor spatial co-expression patterns (Cang and Nie, 2020; Pham et al., 2020; Yuan and Bar-Joseph, 2020; Li et al., 2023), our approach focuses on a broader trend of microenvironment impact. In our CCI analysis of mouse brain data, we found a total of 1235 CCI genes, and interestingly 94% of these genes were not previously recorded in the mouse ligand-receptor genes database from “CellTalkDB” (Shao et al., 2021). The finding highlights the valuable supplementary insights alongside ligand-receptor signaling patterns. Moreover, the flexibility of the regression framework allows for potential expansion to include additional predictors, such as those describing ligand-receptor interactions or other mechanisms. While RECCIPE was initially designed for decomposing cell type-specific signals from spot-level ST data, its adaptability extends seamlessly to similar analyses for single-cell resolution ST data by substituting the proportion of the center spot with a binary indicator denoting the center cell type.

We want to note that the RECCIPE framework does not support the detection of CCIs between cells of the same type, despite the plausibility or even common occurrence of such interactions. This design choice stems from the observation of high correlations between the percentages of a given cell type among neighboring spots in multicellular ST data. These correlations result in predictors of high collinearity in the regression models, posing challenges in discerning the CCI signals between cells of the same type. To avoid the issue of singularity due to high collinearity, we opted to focus on detecting CCIs between different cell types in the RECCIPE regression models, with the aim of ensuring the stability of the coefficient estimates. Despite the inherent challenges associated with multicellular ST data, our RECCIPE framework demonstrates effectiveness in detecting CCI among different cell types, as illustrated in simulation studies.

## Data Availability

Publicly available datasets were analyzed in this study. This data can be found here: https://repo-prod.prod.sagebase.org/repo/v1/doi/locate?id=syn22153884&type=ENTITY.

## Supplementary material

SUPPLEMENTARY FIGURE S1 Distribution of cell count numbers per spot in both real and simulated datasets.**(A,C)** illustrate the inferred cell count numbers for spots with diameters of 55 μm and 100 μm, derived from a real SeqFish+ dataset. **(B,D)** represent the distributions for simulated data under the low- and high-resolution settings respectively.

SUPPLEMENTARY FIGURE S2
**(A)** Spatial distribution for Astro-Ependymal cell proportion of each spot among 8 samples. **(B)** Spatial distribution for Oligodendrocyte lineage and OEG proportion of each spot among 8 samples. **(C)**. Spatial distribution for Vasculature cell proportion of each spot among 8 samples.
