# Supplementary figures and images for: RECCIPE: A new framework assessing localized cell-cell interaction on gene expression in multicellular ST data
Source: Front Genet. 2024 Jan 24;15:1322886. doi: 10.3389/fgene.2024.1322886 (PMC10847567; doi:10.3389/fgene.2024.1322886)

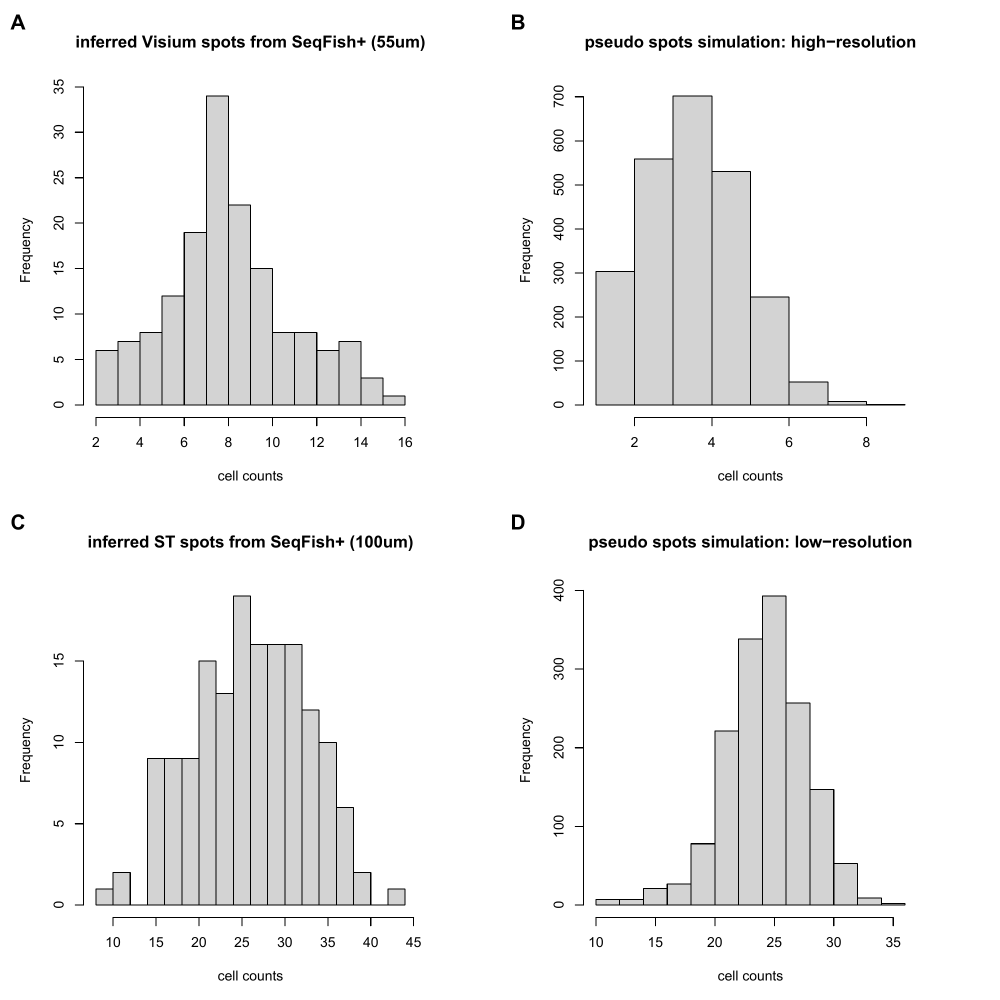

Supplement: Supplementary file 1 [file Image1.TIFF]

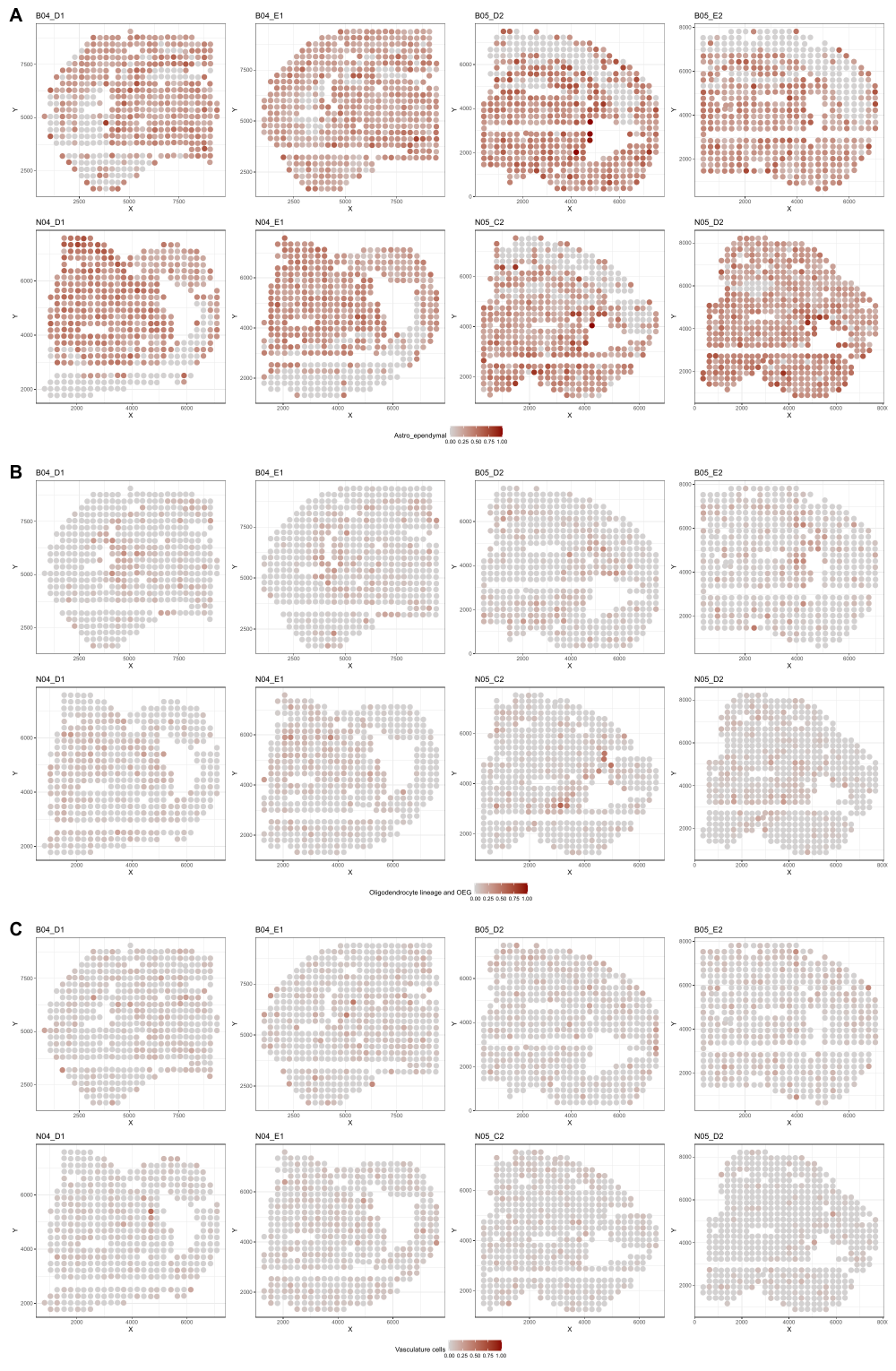

Supplement: Supplementary file 3 [file Image2.TIFF]
